# Using Machine Learning Techniques to Predict Hospital Admission at the Emergency Department

**DOI:** 10.2478/jccm-2022-0003

**Published:** 2022-05-12

**Authors:** Georgios Feretzakis, George Karlis, Evangelos Loupelis, Dimitris Kalles, Rea Chatzikyriakou, Nikolaos Trakas, Eugenia Karakou, Aikaterini Sakagianni, Lazaros Tzelves, Stavroula Petropoulou, Aikaterini Tika, Ilias Dalainas, Vasileios Kaldis

**Affiliations:** 1 Sismanogleio General Hospital, Athens, Greece; 2 Sotiria General Hospital of Chest Diseases of Athens, Athens, Greece; 3 Hellenic Open University, Patras, Greece

**Keywords:** emergency department, emergency medicine, machine learning techniques, artificial intelligence, biomarkers

## Abstract

**Introduction:**

One of the most important tasks in the Emergency Department (ED) is to promptly identify the patients who will benefit from hospital admission. Machine Learning (ML) techniques show promise as diagnostic aids in healthcare.

**Aim of the study:**

Our objective was to find an algorithm using ML techniques to assist clinical decision-making in the emergency setting.

**Material and methods:**

We assessed the following features seeking to investigate their performance in predicting hospital admission: serum levels of Urea, Creatinine, Lactate Dehydrogenase, Creatine Kinase, C-Reactive Protein, Complete Blood Count with differential, Activated Partial Thromboplastin Time, DDi-mer, International Normalized Ratio, age, gender, triage disposition to ED unit and ambulance utilization. A total of 3,204 ED visits were analyzed.

**Results:**

The proposed algorithms generated models which demonstrated acceptable performance in predicting hospital admission of ED patients. The range of F-measure and ROC Area values of all eight evaluated algorithms were [0.679-0.708] and [0.734-0.774], respectively. The main advantages of this tool include easy access, availability, yes/no result, and low cost. The clinical implications of our approach might facilitate a shift from traditional clinical decision-making to a more sophisticated model.

**Conclusions:**

Developing robust prognostic models with the utilization of common biomarkers is a project that might shape the future of emergency medicine. Our findings warrant confirmation with implementation in pragmatic ED trials.

## Introduction

The Emergency Department (ED) represents a key element of any given healthcare facility and retains a high public profile. ED staff manage patients with a huge variety of medical problems and deal with all sorts of emergencies. ED congestion resulting in delays in care remains a frequent issue that prompts the development of tools for rapid triage of high-risk patients [1]. Moreover, it is well documented that timely interventions are critical for several acute diseases [2, 3]. One of the most commonly encountered ED priorities is to quickly identify those who will need hospital admission. Traditionally, this decision relies on clinical judgment aided by the results of laboratory tests. Human factors leading to diagnostic errors occur frequently and are associated with increased morbidity and mortality [4].

Machine Learning (ML) techniques show promise as diagnostic aids in healthcare and have sparked the discussion for their wider application in the ED [5]. Developing robust prognostic models with the utilization of common biomarkers to facilitate rapid and reliable decision-making regarding hospital admission of ED patients is a project that might shape the future of emergency medicine. However, relevant data from the ED is scarce. Recent studies have focused on clinical outcome and mortality prediction [6, 7].

We assessed biochemical markers and coagulation tests that are routinely checked in patients visiting the ED, seeking to investigate their performance in predicting whether the patients will be admitted to the hospital. Our aim is to find an algorithm using ML techniques to assist clinical decision-making in the emergency setting.

## Materials and methods

This research is a retrospective observational study conducted in the ED of a public tertiary care hospital in Greece that has been approved by the Institutional Review Board of Sismanogleio General Hospital (Ref. No 15177/2020, 5969/2021).

This study examines the performance of eight machine learning models based on data of the Biochemistry and Hematology Departments from ED patients. Blood samples were obtained for the measurement of biochemical and hematological parameters. The serum levels of Urea (UREA) [Normal Range (NR)=10-50 mg/dL-test principle: kinetic test with urease and glutamate dehydrogenase], Creatinine (CREA) (NR=0.5-1.5 mg/dL-kinetic colorimetric assay based on the Jaffé method), Lactate Dehydrogenase (LDH) (NR=135-225 U/L-UV assay), Creatine Kinase (CPK) (NR=25-190 U/L-UV assay), C-Reactive Protein (CRP) (NR < 6 mg/L-particle‑enhanced immunoturbidimetric assay) were measured using the Cobas 6000 c501 Analyzer (Roche Diagnostics, Mannheim, Germany). Complete blood count (CBC) samples were collected, and parameters such as White Blood Cell (WBC) (NR=4-11 K/μl-flow cytometry analysis), Neutrophil (NEUT) (NR=40-75 %-flow cytometry), Lymphocyte (LYM) (NR=20-40%-flow cytometry) and Platelet (PLT) (NR=150-400 K/μl-hydrodynamic focusing-flow cytometry) counts and Hemoglobin (HGB) (NR=12-17.5 g/dL-SLS method) were analyzed using the Sysmex XE 2100 Automated Hematology Analyzer (Sysmex Corporation, Kobe, Japan). Routine hemostasis parameters such as activated partial thromboplastin time (aPTT) (NR=24-39 sec-clotting method), DDimer (DD) (NR <500 μg/L-immunoturbidimetric assay), and International Normalized Ratio (INR) (NR=0.86-1.20-calculated) were determined in plasma using the BCS XP Automated Hemostasis Analyzer (Siemens Healthcare Diagnostics, Marburg, Germany).

All raw data was retrieved from a standard Hospital Information System (HIS) and a Laboratory Information System (LIS). The analysis was performed using the Waikato Environment for Knowledge Analysis (WEKA) [8], a Data Mining Software in Java workbench.

**Fig. 1:**
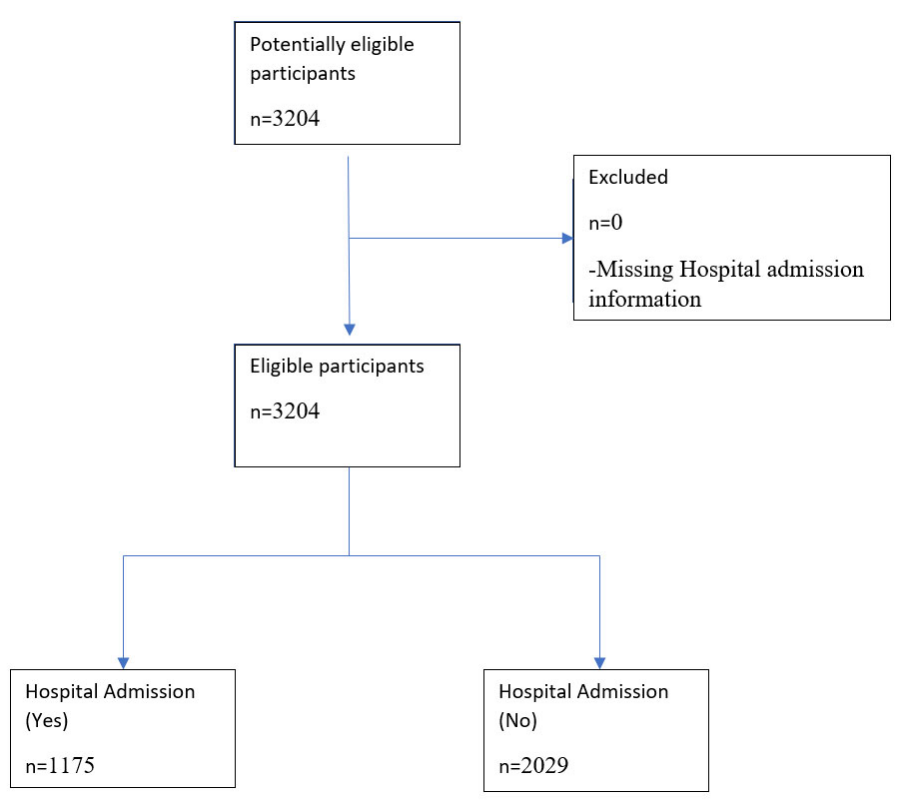
Patient flow diagram

The flow diagram of the study is depicted in Figure 1. A total of 3,204 ED visits were analyzed during the study period (14 March – 4 May 2019). The anonymous data set under investigation contains eighteen features presented in Table 1.

**Table 1:** Features

Features	Type	Mean	Standard Deviation
CPK	numerical	179.155	1183.877
CREA	numerical	1.06	0.827
CRP	numerical	39.094	71.48
LDH	numerical	222.327	156.343
UREA	numerical	45.651	33.616
aPTT	numerical	34.227	11.443
DDIMER	numerical	1422.899	2522.921
INR	numerical	1.131	0.571
HGB	numerical	12.87	2.13
LYM	numerical	22.085	11.672
NEUT	numerical	69.478	13.083
PLT	numerical	252.467	87.814
WBC	numerical	9.617	5.153
Age	numerical; Integer*	61.175	20.822
Gender	categorical {Male, Female}		
ED Unit	categorical {Urology, Pulmonology, Internal Medicine, Otolaryngology, Triage, Cardiology, General Surgery, Opthalmology, Vascular Surgery, Thoracic Surgery}		
Ambulance	Categorical {Yes, No}		
Admission	Categorical {Yes, No}		

*Patients’ age has been rounded to the nearest whole number

To assess the performance of the best-performing model (Smith and Frank 2016) for our analysis in WEKA, we have used a 10-fold cross-validation approach to avoid overfitting; Cross-validation is widely regarded as a quite reliable way to assess the quality of results from machine learning techniques. WEKA [9, 10, 11] provides detailed results for the classifiers under investigation regarding the following evaluation measures:

a. TP Rate (or Recall) is calculated as $\frac{TP}{TP+FN}$

b. FP Rate is calculated as $\frac{FP}{FP+TN}$

c. Precision is calculated as $\frac{TP}{TP+FP}$

d. F-Measure is calculated as $\frac{2\;\;\times \;\;Precision\;\;\times \;\;Recall}{Precision\;\;+\;\;Recall}$

e. MMC is calculated as


$$\frac{TP\times TN-FP\times FN}{\sqrt{\left(TP+FP)\times (TP+FN)\times (TN+FP)\times (TN+FN\right)}}$$

f. The area under the Receiver Operating Characteristics (ROC) curve (AUC)

g. The PRC plot shows the relationship between precision and sensitivity.

Among many algorithms that were evaluated for our research purposes, in this article, we present only the eight best-performing algorithms, mainly in terms of ROC area and F-Measure.

During our experiments, we retained the default settings of all classification algorithms’ original implementations provided by WEKA. Each algorithm was evaluated on two data sets; the original data set, including the missing values, and on the data set where the missing values were identified, and they were replaced with appropriate values using WEKA’s *ReplaceMissing-Values filter*. Furthermore, since the number of patients in our data set who met clinical criteria for hospital admission (36.7%) is less than those who did not meet (63.3%), we applied WEKA’s ClassBalancer technique [8] to prevent overfitting by reweighting the instances in the data set so that each class had the same total weight during the phase of model training.

In our investigation, we evaluated a Naive Bayes classifier [12, 13], a multinomial logistic regression model with a ridge estimator [14], two boosting techniques; AdaBoost [15] and LogitBoost [16], Classification via Regression [17], a random forest [18], a bagging method [19] and a multilayer perceptron (MLP) (a neural network trained with error backpropagation) [8, 20].

## Results

**Fig. 2:**
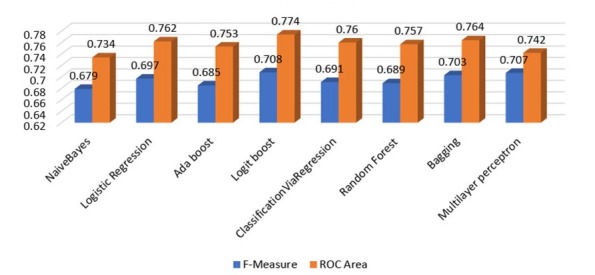
Weighted Average values of F-Measure and ROC Area for all methods (10-fold cross-validation)

The performance of each algorithm was evaluated on its ability to predict whether a patient seen in the emergency department is subsequently admitted to the hospital or not by only taking into consideration the features presented in Table 1. All algorithms were evaluated on both datasets (original with missing values, modified by using the *ReplaceMissingValues* filter), and the detailed results are presented in Appendix (Tables A1-A16). The classification performance’s results on the original data set, regarding the F-Measure and ROC Area of each algorithm, are summarized in Table 2 and Figure 2.

**Table 2:** Weighted Average values of F-Measure and ROC Area for all methods (10-fold cross-validation)

	F-Measure	ROC Area
NaiveBayes	0.679	0.734
Logistic Regression	0.697	0.762
Ada boost	0.685	0.753
Logit boost	0.708	0.774
ClassificationViaRegression	0.691	0.760
Random Forest	0.689	0.757
Bagging	0.703	0.764
Multilayer perceptron	0.707	0.742

According to Table 2, considering the weighted average values, it can be seen that Logit boost slightly outperformed other models with respect to both F-measure and ROC Area with values of 0.708 and 0.774, respectively. We can also observe that the range of F-measure and ROC Area values of all eight algorithms that were evaluated are [0.679-0.708] and [0.734-0.774], respectively, and they can be considered acceptable [21].

**Fig. 3:**
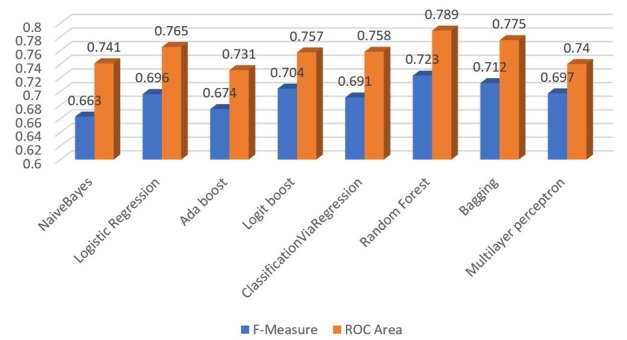
Weighted Average values of F-Measure and ROC Area for all methods - ReplaceMissingValues filters (10-fold cross-validation)

The classification performance’s results of F-Measure and ROC Area on the data set where the missing values have been replaced by using WEKA’s ReplaceMissing-Values filter are summarized in Table 3 and Figure 3.

**Table 3:** Weighted Average values of F-Measure and ROC Area for all methods -ReplaceMissingValues filters (10-fold cross-validation)

	F-Measure	ROC Area
NaiveBayes	0.663	0.741
Logistic Regression	0.696	0.765
Ada boost	0.674	0.731
Logit boost	0.704	0.757
ClassificationViaRegression	0.691	0.758
Random Forest	0.723	0.789
Bagging	0.712	0.775
Multilayer perceptron	0.697	0.740

According to Table 3, considering the weighted average values, it can be seen that the Random Forest slightly outperformed other models with respect to both F-measure and ROC Area with values of 0.723 and 0.789, respectively. Additionally, we can observe that the range of F-measure and ROC Area values of all eight algorithms are [0.663-0.723] and [0.731-0.789], respectively, and as previously noted, they can also be considered acceptable. Furthermore, we were positively surprised to see that the impact of missing values on the classifiers’ performance was less pronounced than we initially thought.

Furthermore, since the admitted patients were 1175 versus 2029 that were not admitted, we applied the WEKA’s ClassBalancer technique on both datasets and re-evaluate the performance of the two classifiers (Logit boost and Random Forest). After the application of the ClassBalancer filter in the original data set, we observe that the performance of Logit boost (F-measure:0.693; ROC area:0.773) (Table A17) is quite similar to this of the imbalanced data set (F-measure:0.708; ROC area:0.774). Similar behavior, we also observe in the performance of Random Forest before (F-measure:0.723; ROC area:0.789) and after (F-measure:0.704; ROC area:0.784) (Table A18) the application of Class-Balancer filter in the data set where the missing values have been replaced by using the ReplaceMissingValues filter.

## Discussion

Based on the data from 3,204 adult ED visits, using common laboratory tests and basic demographics, we evaluated eight ML algorithms that generated models that can reliably predict the hospital admission of patients seen in the ED. Our study utilized pre-existing patient data from a standard HIS and LIS. Therefore, the methods proposed here can serve as a valuable tool for the clinician to decide whether to admit or not an ED patient. The main advantages of this tool include easy access, availability, yes/no result, and low cost. The clinical implications of our approach might be significant and might facilitate a shift from traditional clinical decision-making to a more sophisticated model.

The application of machine learning techniques in the ED is not entirely new. Yet, it is not considered the standard of care. Current efforts are aiming to develop and integrate clinical decision support systems able to provide objective criteria to healthcare professionals. Our study is consistent with previous research showing that logistic regression is the most frequently used technique for model design. The area under the receiver operating curve (AUC) is the most frequently used performance measure [22]. Moreover, the major goal of such predictive tools is to identify high-risk patients accurately and differentiate them from stable, low-risk patients that can be safely discharged from the ED [23] and communicate this identification to the medical expert who can take this information into account while making a decision on admission or discharge.

The hectic pace of work and the stressful setting of the ED have negative consequences on patient safety [24, 25]. It is well established that human factors play an important role in the efficiency of healthcare systems. Different error types have different underlying mechanisms and require specific methods of risk management [26]. A fearful shortcoming for the emergency physician is to fail to admit a seriously ill patient. Our methods might be useful to reduce these errors while explicitly acknowledging that they are meant to aid and not substitute clinical judgment.

In summary, we present an inexpensive clinical decision support tool derived from readily available patient data. This tool is intended to aid the emergency physician regarding hospital admission decisions, as the development of machine learning models represents a rapidly evolving field in healthcare.

## Limitations

This study is not without limitations. In our analysis, we did not include clinical parameters such as the vital signs and the Emergency Severity Index (ESI) [27]. We aimed to investigate whether our model can identify hospital admissions without taking into account clinical data. Thus, we included limited input variables in order to present a low-cost decision support tool with the minimum available data from our HIS. Τhere were also missing values in the data we collected and analyzed; for example, not all of the analyzed ED visits had all the laboratory investigations available. Furthermore, our preliminary findings have not yet been followed up by an implementation phase, and the proposed algorithms have not been validated in a pragmatic ED trial. Therefore, future research is warranted in order to demonstrate whether they can actually improve care.

## Conclusions

In this study, we evaluated a collection of very popular ML classifiers on data from an ED. The proposed algorithms generated models which demonstrated acceptable performance in predicting hospital admission of ED patients based on common biochemical markers, coagulation tests, basic demographics, ambulance utilization, and triage disposition to the ED unit. Our research confirms the prevalent current notion that the utilization of artificial intelligence may have a favorable impact on the future of emergency medicine.

## Appendix

**Table A1:** Performance results by class of NaiveBayes (10-fold cross-validation)

	TP Rate	FP Rate	Precision	Recall	F-Measure	MCC	ROC Area	PRC Area	Admission
	0.360	0.091	0.696	0.360	0.474	0.330	0.734	0.602	Yes
	0.909	0.640	0.710	0.909	0.797	0.330	0.734	0.811	No
Weighted Avg.	0.708	0.439	0.705	0.708	0.679	0.330	0. 734	0.734	

**Table A2:** Performance results by class of NaiveBayes –ReplaceMissingValues filters (10-fold cross-validation)

	TP Rate	FP Rate	Precision	Recall	F-Measure	MCC	ROC Area	PRC Area	Admission
	0.329	0.091	0.677	0.329	0.442	0.300	0.741	0.603	Yes
	0.909	0.671	0.700	0.909	0.791	0.300	0.741	0.822	No
Weighted Avg.	0.696	0.458	0.692	0.696	0.663	0.300	0.741	0.742	

**Table A3:** Performance results by class of Logistic Regression (10-fold cross-validation)

	TP Rate	FP Rate	Precision	Recall	F-Measure	MCC	ROC Area	PRC Area	Admission
	0.456	0.142	0.650	0.456	0.536	0.346	0.762	0.641	Yes
	0.858	0.544	0.732	0.858	0.790	0.346	0.762	0.838	No
Weighted Avg.	0.711	0.396	0.702	0.711	0.697	0.346	0.762	0.766	

**Table A4:** Performance results by class of Logistic Regression–ReplaceMissingValues filters (10-fold cross-validation)

	TP Rate	FP Rate	Precision	Recall	F-Measure	MCC	ROC Area	PRC Area	Admission
	0.456	0.143	0.649	0.456	0.536	0.345	0.765	0.643	Yes
	0.857	0.544	0.731	0.857	0.789	0.345	0.765	0.841	No
Weighted Avg.	0.710	0.397	0.701	0.710	0.696	0.345	0.765	0.768	

**Table A5:** Performance results by class of AdaBoost (10-fold cross-validation)

	TP Rate	FP Rate	Precision	Recall	F-Measure	MCC	ROC Area	PRC Area	Admision
	0.423	0.137	0.642	0.423	0.510	0.323	0.753	0.620	Yes
	0.863	0.577	0.721	0.863	0.786	0.323	0.753	0.836	No
Weighted Avg.	0.702	0.415	0.692	0.702	0.685	0.323	0.753	0.757	

**Table A6:** Performance results by class of AdaBoost–ReplaceMissingValues filters (10-fold cross-validation)

	TP Rate	FP Rate	Precision	Recall	F-Measure	MCC	ROC Area	PRC Area	Admision
	0.396	0.133	0.634	0.396	0.487	0.302	0.731	0.604	Yes
	0.867	0.604	0.713	0.867	0.782	0.302	0.731	0.815	No
Weighted Avg.	0.694	0.431	0.684	0.694	0.674	0.302	0.731	0.738	

**Table A7:** Performance results by class of LogitBoost (10-fold cross-validation)

	TP Rate	FP Rate	Precision	Recall	F-Measure	MCC	ROC Area	PRC Area	Admission
	0.489	0.147	0.658	0.489	0.561	0.370	0.774	0.657	Yes
	0.853	0.511	0.742	0.853	0.794	0.370	0.774	0.854	No
Weighted Avg.	0.719	0.377	0.711	0.719	0.708	0.370	0.774	0.782	

**Table A8:** Performance results by class of LogitBoost –ReplaceMissingValues filters (10-fold cross-validation)

	TP Rate	FP Rate	Precision	Recall	F-Measure	MCC	ROC Area	PRC Area	Admission
	0.464	0.135	0.666	0.464	0.547	0.364	0.757	0.641	Yes
	0.865	0.536	0.736	0.865	0.795	0.364	0.757	0.837	No
Weighted Avg.	0.718	0.389	0.710	0.718	0.704	0.364	0.757	0.765	

**Table A9:** Performance results by class of ClassificationViaRegression (10-fold cross-validation)

	TP Rate	FP Rate	Precision	Recall	F-Measure	MCC	ROC Area	PRC Area	Admission
	0.447	0.145	0.641	0.447	0.527	0.334	0.760	0.639	Yes
	0.855	0.553	0.727	0.855	0.786	0.334	0.760	0.839	No
Weighted Avg.	0.705	0.403	0.696	0.705	0.691	0.334	0.760	0.766	

**Table A10:** Performance results by class of ClassificationViaRegression–ReplaceMissingValues filters (10-fold cross-validation)

	TP Rate	FP Rate	Precision	Recall	F-Measure	MCC	ROC Area	PRC Area	Admission
	0.447	0.145	0.641	0.447	0.527	0.334	0.758	0.638	Yes
	0.855	0.553	0.727	0.855	0.786	0.334	0.758	0.837	No
Weighted Avg.	0.705	0.403	0.696	0.705	0.691	0.334	0.758	0.764	

**Table A11:** Performance results by class of Random Forest (10-fold cross-validation)

	TP Rate	FP Rate	Precision	Recall	F-Measure	MCC	ROC Area	PRC Area	Admission
	0.394	0.103	0.689	0.394	0.501	0.345	0.757	0.650	Yes
	0.897	0.606	0.719	0.897	0.798	0.345	0.757	0.832	No
Weighted Avg.	0.713	0.422	0.708	0.713	0.689	0.345	0.757	0.765	

**Table A12:** Performance results by class of Random Forest–ReplaceMissingValues filters (10-fold cross-validation)

	TP Rate	FP Rate	Precision	Recall	F-Measure	MCC	ROC Area	PRC Area	Admission
	0.540	0.161	0.660	0.540	0.594	0.399	0.789	0.676	Yes
	0.839	0.460	0.759	0.839	0.797	0.399	0.789	0.858	No
Weighted Avg.	0.729	0.350	0.723	0.729	0.723	0.399	0.789	0.791	

**Table A13:** Performance results by class of Bagging (10-fold cross-validation)

	TP Rate	FP Rate	Precision	Recall	F-Measure	MCC	ROC Area	PRC Area	Admission
	0.471	0.142	0.657	0.471	0.549	0.360	0.764	0.654	Yes
	0.858	0.529	0.737	0.858	0.793	0.360	0.764	0.840	No
Weighted Avg.	0.716	0.387	0.708	0.716	0.703	0.360	0.764	0.772	

**Table A14:** Performance results by class of Bagging–ReplaceMissingValues filters (10-fold cross-validation)

	TP Rate	FP Rate	Precision	Recall	F-Measure	MCC	ROC Area	PRC Area	Admission
	0.515	0.161	0.649	0.515	0.574	0.375	0.775	0.654	Yes
	0.839	0.485	0.749	0.839	0.791	0.375	0.775	0.852	No
Weighted Avg.	0.720	0.366	0.712	0.720	0.712	0.375	0.775	0.779	

**Table A15:** Performance results by class of Multilayer perceptron (10-fold cross-validation)

	TP Rate	FP Rate	Precision	Recall	F-Measure	MCC	ROC Area	PRC Area	Admission
	0.542	0.190	0.623	0.542	0.580	0.364	0.742	0.622	Yes
	0.810	0.458	0.753	0.810	0.781	0.364	0.742	0.815	No
Weighted Avg.	0.712	0.360	0.705	0.712	0.707	0.364	0.742	0.744	

**Table A16:** Performance results by class of Multilayer perceptron–ReplaceMissingValues filters (10-fold cross-validation)

	TP Rate	FP Rate	Precision	Recall	F-Measure	MCC	ROC Area	PRC Area	Admission
	0.480	0.161	0.633	0.480	0.546	0.343	0.740	0.617	Yes
	0.839	0.520	0.736	0.839	0.784	0.343	0.740	0.808	No
Weighted Avg.	0.707	0.388	0.698	0.707	0.697	0.343	0.740	0.738	

**Table A17:** Performance results by class of Logit Boost– ClassBalancer filter (10-fold cross-validation)

	TP Rate	FP Rate	Precision	Recall	F-Measure	MCC	ROC Area	PRC Area	Admission
	0.685	0.300	0.696	0.685	0.690	0.385	0.773	0.758	Yes
	0.700	0.315	0.690	0.700	0.695	0.385	0.773	0.779	No
Weighted Avg.	0.693	0.307	0.693	0.693	0.693	0.385	0.773	0.769	

**Table A18:** Performance results by class of Random Forest– ReplaceMissingValues and ClassBalancer filters (10-fold cross-validation)

	TP Rate	FP Rate	Precision	Recall	F-Measure	MCC	ROC Area	PRC Area	Admission
	0.653	0.243	0.729	0.653	0.689	0.412	0.784	0.767	Yes
	0.757	0.347	0.686	0.757	0.720	0.412	0.784	0.783	No
Weighted Avg.	0.705	0.295	0.707	0.705	0.704	0.412	0.784	0.775	
